# Femorocele: A Rare Form of Femoral Hernia

**DOI:** 10.70352/scrj.cr.25-0491

**Published:** 2025-10-10

**Authors:** Kimihiko Nakamura, Hisashi Kanoda

**Affiliations:** Department of Surgery, Kanto Central Hospital of the Mutual Aid Association of Public School Teachers, Tokyo, Japan

**Keywords:** femorocele, femoral hernia, groin swelling, cystic sac, hydrocele

## Abstract

**INTRODUCTION:**

Femorocele, also known as a hydrocele of the femoral hernia (FH) sac, is a rare form of FH. Initial misdiagnosis is common not only in FH but is even more frequent in femorocele, due to its rarity and non-distinctive features that make preoperative recognition challenging; in many cases, femorocele is identified only intraoperatively or postoperatively. This report describes a case of femorocele that initially posed a diagnostic challenge and was initially misdiagnosed as a ganglion.

**CASE PRESENTATION:**

A 63-year-old woman presented with a right sided groin swelling which was suspected as ganglion at the other department. Physical examination was notable for non-reducible and painless mass lateral to pubic tubercle. CT revealed a hydrocele, measuring 57.4 mm in max, medial to common femoral vessels and caudal to inguinal ligament, mostly suspicious of femorocele. Elective surgery via the anterior approach revealed no abnormality in the inguinal canal. A hydrocele originating from the peritoneum and protruding into the femoral canal was observed, consistent with a diagnosis of femorocele. The hernia sac was reduced into the abdominal cavity, and the hernia was repaired using a mesh placed in the preperitoneal space, covering the myopectineal orifice. The patient had an uneventful recovery and was discharged on POD 3.

**CONCLUSIONS:**

Femorocele is a rare clinical entity presenting with nonspecific symptoms. Its imaging findings require careful interpretation to avoid misdiagnosis. Surgery remains the only means for establishing a definitive diagnosis and providing curative treatment. Since most cases are identified intraoperatively or postoperatively, or initially misdiagnosed as other conditions, it is essential that all clinicians remain aware of this rare entity.

## Abbreviations

CCN
: cyst of the canal of Nuck
FH
: femoral hernia

## INTRODUCTION

FH is characterized by protrusion of the peritoneum through the femoral ring, with the hernia sac descending into the femoral canal inferior to the inguinal ligament.^1)^ Therefore, a groin swelling below the inguinal ligament is typically diagnosed as an FH, while one above it suggests an inguinal hernia.^1)^ As FHs account for only 2%–8% of all groin hernias and occur in a similar anatomical location, they are sometimes misdiagnosed as inguinal hernias or other conditions, making accurate diagnosis challenging.^2)^ Femorocele, also known as a hydrocele of the FH sac, is a rare form of FH characterized by a fluid collection in the sac. Due to its rarity and the difficulty in diagnosis, most cases of femorocele are diagnosed intraoperatively or postoperatively, or are initially misdiagnosed as other conditions.^3)^ Herein, we present the unique case of a 63-year-old woman with groin swelling, initially suspected to be a ganglion, and ultimately diagnosed with femorocele and treated via anterior approach.

## CASE PRESENTATION

**Fig. 1:**
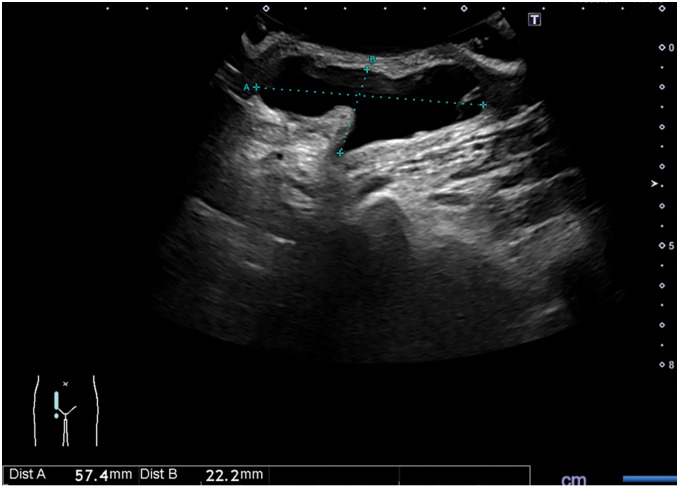
Ultrasonography of the right groin. A cystic mass with low-level internal echoes and no blood flow on color Doppler was observed, measuring 57.4 mm in maximum diameter.

**Fig. 2:**
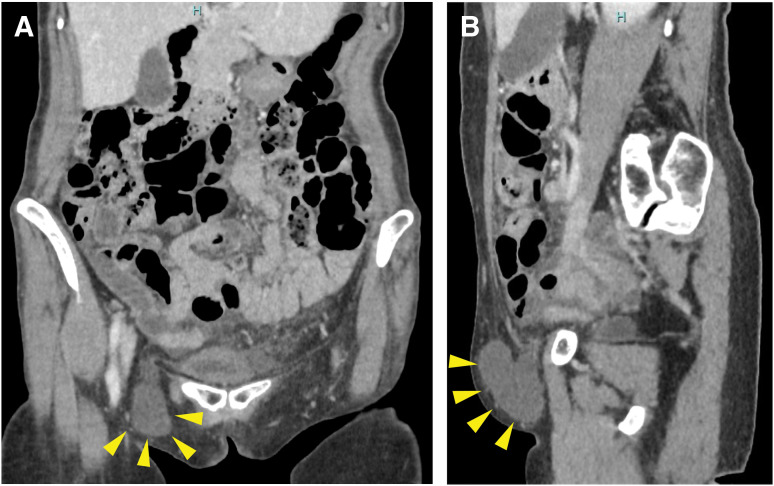
Contrast-enhanced CT of the abdomen. Showing a hydrocele in the groin, measuring 57.4 × 57.0 × 22.2 mm, medial to common femoral vessels and caudal to inguinal ligament (arrowheads). (**A**) Coronal section; (**B**) Sagittal section.

A 63-year-old woman presented with a 2-week history of right-sided groin swelling which was suspected as ganglion at the other department. She had a recent history of suspected localized scleroderma, and was on 30 mg of prednisolone with favorable response. She was 153.9 cm tall, weighed 41.1 kg, and had a BMI of 17.35. The vital signs were normal and the physical examination was notable for a non-reducible and painless mass lateral to the pubic tubercle. Ultrasonography of the right groin revealed a cystic mass with low-level internal echoes and no blood flow on color Doppler, measuring 57.4 mm in maximum diameter (**Fig. 1**). CT revealed a hydrocele, measuring 57.4 × 57.0 × 22.2 mm, medial to common femoral vessels and caudal to inguinal ligament, mostly suspicious of hydrocele of femoral hernial sac also known as femorocele (**Fig. 2**). Given the patient’s stable condition, elective surgery was performed 1 month after the initial consultation, following tapering of prednisolone to 10 mg. The operation was undertaken to prevent progression to an incarcerated FH and to avoid the potential need for future emergency surgery.

**Fig. 3:**
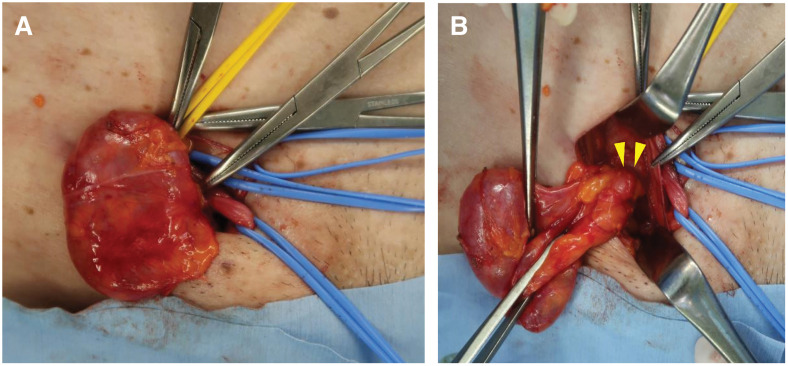
Surgical findings. Dissected cystic sac traced upto the right femoral ring. (**A**) Showing cystic sac. (**B**) Showing cystic sac protruding from the femoral canal (arrowheads), viewed from the caudal aspect of the femoral canal.

Because this was the first case, an anterior approach was chosen to manage any potential difficulties. The inguinal canal was dissected and intraoperative findings revealed no abnormalities within the canal. Upon incising the posterior wall of the inguinal canal, a hydrocele was identified originating from the peritoneum and protruding into the femoral canal. The tip of the hernia sac was also visualized from the caudal aspect of the femoral canal, which was compatible with the diagnosis of femorocele (**Fig. 3**). By expanding the femoral ring without incising the lacunal ligament, the ascites of the hydrocele was gently reduced into the abdominal cavity, followed by reduction of the hernia sac. The hernia was then repaired using a Modified ONFLEX mesh (BD, Franklin Lakes, NJ, USA), which was inserted via the internal inguinal ring and placed in the preperitoneal space to cover the myopectineal orifice, including the femoral ring. The mesh was fixed by suturing the strap to the internal oblique tendon and the inguinal ligament. The operation time was 96 minutes and estimated blood loss was 5 g. The postoperative course was uneventful, and the patient was discharged 3 days after the operation. No recurrence or complications were noted at the 1-month postoperative follow-up, and the patient continues to be under follow-up.

## DISCUSSION

Femorocele, also known as hydrocele of the FH sac is a rare form of FH, characterized by a fluid collection within the hernia sac. It was first described by Sir Astley Cooper, and only a handful of cases have been documented worldwide.^3)^ Femorocele can be classified into two types. Primary femorocele occurs when fluid accumulates in the sac of an FH due to obstruction of its communication with the peritoneal cavity, in the absence of ascites. This obstruction may result from an omental plug or adhesions at the narrow neck of the femoral ring, or from complete obliteration caused by traumatic or inflammatory processes, in which hemorrhage or inflammatory exudates lead to fusion of the serous surfaces. Secondary femorocele refers to fluid collection in the sac of a femoral hernia that arises from the peritoneal cavity, usually due to ascites accumulating in the hernia sac.^4–6)^ In the present case, there was no evidence of ascites on CT, and no history of heart failure or cirrhosis was noted making secondary femorocele unlikely. Although no definitive cause of primary femorocele was identified, an omental plug or adhesions might have caused obstruction of its communication with the peritoneal cavity.

The clinical presentation of femorocele is cystic, soft and non-tender, irreducible swelling below the inguinal ligament like in our case.^4)^ On physical examination, femorocele may be differentiated from a typical FH by the presence of a gradually enlarging, fluid-filled sac that may be transilluminated, is irreducible within the femoral canal, and lacks a cough impulse.^4)^ Although ultrasonography is a convenient and noninvasive modality, the best diagnostic modality is CT, also providing a clear road map for planning the surgical procedure.^5)^ Although a cystic groin mass on imaging is often indicative of the relatively more common diagnosis of a CCN, in our case a ganglion was also considered.^6)^ The differential diagnosis for such a condition in the groin will be irreducible or incarcerated FH, CCN, subcutaneous lipoma, Bartholin’s cyst, lymphadenopatheic abscess, or arterial and venous aneurysms.^1)^ In the present case, ultrasonography had been performed prior to consultation to our department by a laboratory technician who was not familiar with femorocele. Therefore, bedside ultrasonography at the time of consultation might have increased our confidence in establishing the diagnosis.

Surgery is the only radical treatment to prevent the recurrence and future development of an FH.^3)^ Although Malaibari and Niebuhr^7)^ was the first to report a case of laparoscopic treatment of femorocele, in which the hernia sac was successfully extracted and mesh was placed using the transabdominal preperitoneal repair technique, there is currently no established recommendation for the optimal surgical approach to treat femorocele. For FH, an endoscopic approach is generally recommended in elective repair because it is associated with fewer recurrences compared with open repair.^8)^ However, the anterior approach may be preferable in the following cases: 1. When the laparoscopic approach is technically challenging, such as in cases of a femoral hernia sac filled with fluid and dissected through a narrowed femoral ring.^5)^ 2. In cases of infected femorocele, as reported by Bakshi,^9)^ mesh repair is generally considered relatively contraindicated, and primary suture repair may be challenging via the laparoscopic approach. 3. If the case turns out to be a CCN, and the lesion extends above the pubic bone or toward the distal portion of the round ligament near the labia majora, safe resection via the intraperitoneal approach becomes difficult.^10)^ 4. (Partly overlaps with reasons 1. and 3.) When the diagnosis is uncertain or laparoscopic techniques are not sufficiently reliable, and management of potential intraoperative difficulties is anticipated. As in the present case, this was our first experience with femorocele, and although we carefully interpreted the CT findings, the diagnosis remained unconfident.

Because of its rarity, there are currently no established recommendations regarding the use of mesh. Although several reported cases of femorocele have described suturing the inguinal ligament to the iliopectineal ligament to close the femoral ring without tension, the use of mesh is considered safe and carries a low risk of complications in uncomplicated cases.^6)^ Given that mesh repair via a preperitoneal approach is effective and associated with lower rates of recurrence and postoperative pain than mesh plug repair for femoral hernia, we adopted the preperitoneal approach in our case.^11)^

Due to its rarity and diagnostic difficulty, most cases are identified intra- or postoperatively, or misdiagnosed as other conditions—such as a ganglion in our case. Awareness of this condition is important not only for surgeons but also for all clinicians, including physicians and radiologists.

## CONCLUSIONS

Femorocele is a rare clinical entity that may present with nonspecific symptoms. Its imaging findings should be interpreted with careful attention to their positional relationship with surrounding structures to avoid misdiagnosis. Although its pathophysiology is not emergent, surgery remains the only means of achieving both a reliable diagnosis and curative treatment. As most cases are identified intra- or postoperatively or misdiagnosed as other conditions, all clinicians should be aware of this rare condition.
